# Fatty liver is associated with blood pathways of inflammatory response, immune system activation and prothrombotic state in Young Finns Study

**DOI:** 10.1038/s41598-018-28563-y

**Published:** 2018-07-09

**Authors:** Tuukka Taipale, Ilkka Seppälä, Emma Raitoharju, Nina Mononen, Leo-Pekka Lyytikäinen, Thomas Illig, Melanie Waldenberger, Markus Juonala, Nina Hutri-Kähönen, Niku Oksala, Mika Kähönen, Olli Raitakari, Terho Lehtimäki

**Affiliations:** 1 Department of Clinical Chemistry, Fimlab Laboratories and Finnish Cardiovascular Research Center - Tampere, Faculty of Medicine and Life Sciences, University of Tampere, Tampere, Finland; 2 Department for Human Genetics, Hannover Medical School, Hannover, Germany; 3 Hannover Unified Biobank, Hannover Medical School, Hannover, Germany; 4 Research Unit of Molecular Epidemiology, Institute of Epidemiology II, Helmholtz Zentrum München, German Research Center for Environmental Health, Neuherberg, Germany; 5 Department of Medicine, University of Turku, Turku, Finland; 6 Division of Medicine, Turku University Hospital, Turku, Finland; 7 Department of Pediatrics, Tampere University Hospital and Faculty of Medicine and Life Sciences, University of Tampere, Tampere, Finland; 8 Division of Vascular Surgery, Department of Surgery, Tampere University Hospital, Tampere, Finland; 9 Department of Clinical Physiology, Tampere University Hospital and Finnish Cardiovascular Research Center - Tampere, Faculty of Medicine and Life Sciences, University of Tampere, Tampere, Finland; 10 Research Centre for Applied and Preventive Cardiovascular Medicine, University of Turku, Turku, Finland; 11 Department of Clinical Physiology and Nuclear Medicine, University of Turku and Turku University Hospital, Turku, Finland

## Abstract

Fatty liver (FL) disease is the most common type of chronic liver disease. We hypothesized that liver’s response to the process where large droplets of triglyceride fat accumulate in liver cells is reflected also in gene pathway expression in blood. Peripheral blood genome wide gene expression analysis and ultrasonic imaging of liver were performed for 1,650 participants (316 individuals with FL and 1,334 controls) of the Young Finns Study. Gene set enrichment analysis (GSEA) was performed for the expression data. Fourteen gene sets were upregulated (false discovery rate, FDR < 0.05) in subjects with FL. These pathways related to extracellular matrix (ECM) turnover, immune response regulation, prothrombotic state and neural tissues. After adjustment for known risk factors and biomarkers of FL, we found i) integrin A4B1 signaling, ii) leukocyte transendothelial migration, iii) CD40/CD40L and iv) netrin-1 signaling pathways to be upregulated in individuals with FL (nominal p < 0.05). From these all but not ii) remained significantly upregulated when analyzing only subjects without history of heavy alcohol use. In conclusion, FL was associated with blood gene sets of ECM turnover, inflammatory response, immune system activation and prothrombotic state. These may form a systemic link between FL and the development of cardiovascular diseases.

## Introduction

The fatty liver disease (FLD) is a common liver disorder in western industrialized countries and an emerging problem in the Asia-Pacific region. FLD is often associated excessive alcohol consumption (alcoholic fatty liver disease, AFLD) or obesity with or without insulin resistance i.e., non-alcoholic fatty liver disease (NAFLD)^1,2^. Currently NAFLD is the most common cause of chronic liver disease^3^, affecting up to one third of US population^4^ and 70–90% of the obese and diabetics^5^. The main characteristic of FLD irrespective of the cause is the accumulation of triglyceride lipid droplets (>5% of liver weight) in liver cells^1–3,6^. The increased level of intrahepatic fatty acids may lead into cell damage and inflammation and provide a source of oxidative stress promoting fatty liver progression from steatosis to steatohepatitis and subsequently to cirrhosis or ultimately to hepatocellular carcinoma^2,6^.

The primary causes leading to hepatocellular lipid accumulation are not yet well understood, but they are thought to include alterations in the hepatic lipid uptake, synthesis, degradation and secretion. Also, its metabolic, systemic and clinical consequences are still incompletely understood^6,7^. For example, clinically patients who eventually develop progressive liver cirrhosis or liver failure cannot be differentiated from those who do not. Immune system activation and inflammation are key players in the pathogenesis of FLD^8–11^. NAFLD is considered as an early mediator of systemic disease^12^. Innate immune system is deeply involved in pathophysiological events of fatty liver by following mechanisms: TLR-4 dependent signaling activates the Kupffer cells, complement pathway activation, balancing the cytokine network towards pro-inflammatory mediators, alternation in natural killer (NK) and NK T cell number and activity, and activation of the adaptive immune system leading to severe liver disease^8^. The involvement of adaptive immunity in the evolution of fatty liver and its complications is under research. Present evidence suggests that adaptive immunity contributes not only to the maintenance of fatty liver but also to the progression and comorbidities of it^8,13^. Immuno-inflammatory mechanisms are present in several comorbidities related with FLD, including obesity, type 2 diabetes, chronic kidney disease, metabolic syndrome, and cardiovascular diseases^5,12^. However, the actual pathophysiological mechanisms connecting these states are not well known.

The purpose of the present study was to reveal alterations in gene pathways related to FLD in a population based study cohort. We hypothesized that accumulated fatty acids and large droplets of triglycerides in liver cells trigger an inflammatory response in liver, which is visible via differential immuno-inflammatory gene pathway expression in blood. Understanding the pathogenic mechanisms of FLD and its metabolic and systemic consequences will give us new insight to the disease process and comorbidities of FLD.

## Material and Methods

### Subjects

The Cardiovascular Risk in Young Finns Study (YFS) is a Finnish longitudinal population study on the evolution of cardiovascular risk factors from childhood to adulthood. These subjects for the baseline study in 1980 were randomly selected from Finnish national registry from six different age groups between 3 to 18 years and five different study districts^14^. In the present study, we used the data from the follow-up in 2011, when 2,063 subjects participated in blood sampling and in clinical examinations. Participants in the follow-up studies have been found to be more often women and older than those who dropped out, but no significant differences in risk factors have been found^14^. The present study has been approved by the Ethics Committee of the Hospital District of Southwest Finland on September 21st, 2010. The study protocol of each study phase corresponded to the proposal by the World Health Organization. All subjects gave written informed consent and the study was conducted in accordance with the Helsinki declaration.

### Clinical examinations

Waist and hip circumference, height and weight were measured and body mass index (BMI) calculated as kg/m². Blood pressure was measured as the average of three measurements taken at 2-minute intervals in a sitting position from the right arm brachial artery by random zero sphygmomanometer. The metabolic syndrome was defined according to the harmonized definition^15^.

Data regarding daily alcohol consumption, physical activity, and smoking were obtained by questionnaires in 2011. Alcohol consumption data were acquired by standardized questionnaires and calculated in standard doses (12 g pure ethanol) per day by dividing the total number of doses consumed per week (0.33 L doses of beer or cider, 0.12 L doses of wine, and 0.04 L doses of hard liquor) by 7. Physical activity index was calculated (range 5–15)^16^. The participants were classified as smokers if they smoked daily. Subjects were classified as diabetics based on questionnaires, blood fasting glucose and glycated hemoglobin measurements and national medication registry records. Complete information on history of HIV and hepatitis C were obtained from the Finnish National Hospital Discharge Register. None of the participants had a diagnosis of hepatitis C or HIV, which could potentially have contributed to the diagnosis of fatty liver.

### Ultrasound imaging of liver

Ultrasound imaging of the liver was performed for 2,040 study participants using a validated protocol^17^ and Sequoia 512 ultrasound mainframes (Acuson, Mountain View, CA, USA) with 4.0 MHz adult abdominal transducers. Evaluation of hepatic steatosis was performed according to liver-to-kidney contrast, parenchymal brightness, deep beam attenuation, and bright vessel walls^18^. According to these criteria the presence of hepatic steatosis was assessed visually from images by a highly trained ultrasonographer. The participants were classified into fatty liver (FL) and normal liver (NL) groups.

### Blood samples and biochemistry

Venous blood samples were drawn from the right antecubital vein after an overnight fast and anticoagulated with EDTA. For liver enzyme quantification serum was separated, aliquoted, and stored at −70 °C until analysis. Serum alanine aminotransferase (ALT), aspartate aminotransferase (AST), gamma-glutamyltransferase (GT) and triglyceride concentrations were measured by enzymatic methods (ALT, AST, GT and Triglycerides System Reagent, Beckman Coulter Biomedical, Ireland). Apolipoprotein B (ApoB) and C-reactive protein (CRP) were determined immunoturbidimetrically (ApoB assay reagent, Orion Diagnostica, Finland and CRP Latex reagent, Beckman Coulter Biomedical). The serum triglyceride concentration was assayed using the enzymatic glycerol kinase–glycerol phosphate oxidase method (Triglyceride reagent, Beckman Coulter Biomedical). All the above-mentioned assays were performed on an automatic analyzer (AU400, Olympus, Japan).

### RNA isolation and quality control

Whole blood (2.5 ml) was collected into PaXgene Blood RNA Tubes (PreAnalytix). The tubes were inverted 8–10 times then stored at room temperature for at least 2 hours. The tubes were frozen (−80 °C) and thawed overnight before RNA isolation (both miRNA and total RNA) with a PAXgene Blood microRNA Kit (Qiagen) including the DNase Set using the QiaCube (Qiagen). The concentrations and purity of the RNA samples were evaluated spectrophotometrically (BioPhotomer, Eppendorf). The RNA isolation process was validated by analyzing the integrity of several RNAs (n = 26) with the RNA 6000 Nano Chip Kit (Agilent).

### Genome wide expression analysis

2,049 individuals gave blood samples to RNA isolation in the 30-year follow-up in 2011–2012. 63 samples were discarded during the RNA isolation protocol, leading to 1,987 samples including 1 technical replicate taking part in the genome wide expression analysis. 322 samples had too low concentration after amplification step. Finally, 1,667 samples were run with the expression BeadChip including 3 technical replicates (2 from the mRNA amplification step).

The expression levels were analyzed with an Illumina HumanHT-12 version 4 Expression BeadChip (Illumina Inc.). In brief, 300–500 ng of RNA was reverse transcribed into cDNA and biotin-UTP labelled using the Illumina TotalPrep RNA Amplification Kit (Ambion), and 1,500 ng of cDNA was then hybridized to the Illumina HumanHT-12 v4 Expression BeadChip. The BeadChips were scanned with the Illumina iScan system.

### Processing of GWE microarray data

Raw Illumina summary probe-level data was exported from Beadstudio and analyzed in R (http://www.r-project.org/) using the Bioconductor (http://www.bioconductor.org/) packages. The HT-12 v4 BeadChip contains 47,231 expression and 770 control probes. The transcripts detected (detection p-value < 0.01) in less than 5% of samples were excluded from the analysis. After this filtering 19,637 genes were used for analysis. We disregarded 4 samples with less than 6,000 significantly detected expression probes (detection p-value < 0.01). The expression data was processed using nonparametric background correction, followed by quantile normalization with control and expression probes, and log2 transformation using the *neqc* function in the *limma* package. Based on *RPS4Y1–2* and *XIST* mRNA levels on the Y and X chromosomes, respectively, we excluded 9 samples due to mismatch with the recorded sex. After quality control, expression data were available for 1,654 samples including 4 technical replicates, which were used to examine batch effects and excluded subsequently. 1,650 non-related samples were used for further analysis.

### Definition of the cases and controls and statistical analysis for the demographics of the study population

Subjects were classified into two groups: FL (n = 316) and NL (n = 1,334) by a highly trained ultrasonographer and FL cases were considered as alcoholic fatty liver (AFL) (n = 55) if the daily alcohol consumption exceeded 20 g for women and 30 g for men^19^. If the alcohol consumption was lower than these thresholds, they were classified into non-alcoholic fatty liver (NAFL) group (n = 223). Demographics of the study participants were analyzed with R (version 3.2.2). These include all the parameters shown to be connected with fatty liver in a previous study of YFS participants including age, sex, BMI, alcohol consumption, smoking, systolic and diastolic blood pressure, waist to hip ratio, ApoB, triglycerides, insulin, liver enzymes (ALT, AST, GT) and physical activity index^20^. Data are presented as mean (SD). P values < 0.05 were considered significant. FL, NAFL and AFL groups were separately compared to control group using Mann-Whitney test for continuous parameters and χ2 test for classified parameters.

### Gene set enrichment analysis (GSEA) of GWE data

The Canonical Pathways (CP) from the curated gene sets collection (C2) of the molecular signature database (MSigDB v5.0) database^21^ were used in gene set enrichment analysis (GSEA). Only pathways with 15 to 500 genes were included, resulting in using altogether 870 pathways in the analysis. The alternative probes representing the same gene in the beadchip were collapsed to single genes by GSEA software, resulting in 14403 genes in the analysis.

GSEA software^22,23^ was used to analyze the association of gene pathways with the phenotype. A false discovery rate (FDR) q-value < 0.25 can be considered as a significant according to the criteria recommended by Subramanian *et al*.^22^. However, we used a more stringent FDR q-value < 0.05 to select the statistically significant pathways. GSEA performs 1000 permutations when calculating the FDR values. Therefore, in the result tables we present p < 0.001 instead of 0 when the calculated FDR value appears as 0 in the GSEA results. R language (version 3.2.2) was used for adjusting the gene expression data before the analysis with GSEA software. The expression levels were adjusted for sex and age (Model 1) and with those independent confounding factors previously associated with FL in our study sample (fully adjusted Model 2)^20^: adjusting the gene expression for age, sex, BMI, alcohol consumption, ApoB, triglycerides, insulin, systolic blood pressure, ALT, and physical activity index. In addition, to reduce the effect of technical factors^24^ we adjusted the expression levels for the first 20 principal components as described elsewhere^25^. Finally, inverse normal transformation was applied to the probes’ residual variance before analyzing them with the GSEA algorithm.

Similar analysis setup using the MSigDB canonical pathways were performed for all measurements: FL vs. NL, NAFL vs. NL and AFL vs. NL. In addition, GSEA was performed for NAFL vs. AFL to reveal the differences in gene expression pathways between these states.

### Data Availability

The datasets analysed during the current study are not publicly available due to the regulations of Finnish law and the ethical permissions that prohibits uploading the YFS data to public platforms.

## Results

### General characteristics of the study sample

General demographics of the study population is presented in Table 1. Individuals with FL were more often men and older and were metabolically less healthy. They also had higher BMI and waist to hip ratio when compared to the NL group. 1.3% of the NL group had diabetes but for FL group the prevalence of diabetes was ten-fold (13.1%). Elevated systolic and diastolic blood pressure, ApoB, triglycerides, insulin, increased levels of liver enzymes (ALT, AST, GT) and CRP were also associated with FL. In addition, alcohol consumption and decreased physical activity were significantly related to FL. All the mentioned characteristics differed significantly (P < 0.001) between the groups. Only exception to the previously identified risk factors^20^ was that daily smoking did not vary from the NL in the FL or NAFL groups in our study. However, smoking differed significantly (p = 0.022) between the subjects with AFL and NL. In addition, in the NAFL subgroup alcohol consumption was similar to the NL group. The subjects in the present study are relatively young and include only 11 individuals with cardiovascular diseases including heart failure, coronary artery disease, coronary artery bypass grafting, coronary angioplasty, cerebrovascular disease and stroke. However, the number of subjects with cardiovascular diseases is insufficient to provide significant pathway enrichment results for this group.

**Table 1:** Summary of the 1,650 study participants: classified into normal liver and fatty liver groups of FL, NAFL and AFL.

	NL	FL	NAFL	AFL
Number of subjects in group	1,334	316	223	55
Age, years	41.55 (5.07)	43.16 (4.73)	42.91 (4.83)	44.47 (4.32)
Males (%)	534 (40.0)	221 (69.9)	151 (67.7)	42 (76.4)
Body mass index, kg/m^2^	25.60 (4.37)	30.75 (5.43)	30.99 (5.64)	30.01 (5.17)
Waist to hip ratio	0.88 (0.08)	0.98 (0.07)	0.98 (0.08)	0.99 (0.08)
Daily smokers (%)	172 (13.5)	48 (16.6)	34 (15.3)	14 (25.5)
Diabetics (%)	22 (1.7)	41 (13.1)	28 (12.7)	8 (14.5)
Metabolic syndrome (%)	167 (12.7)	183 (59.0)	130 (59.4)	33 (62.3)
Systolic BP, mmHg	117.38 (13.60)	128.22 (14.20)	126.89 (13.09)	132.61 (14.82)
Diastolic BP, mmHg	73.56 (9.86)	82.38 (10.64)	82.01 (10.61)	83.40 (10.89)
Apolipoprotein B, g/L	1.01 (0.26)	1.23 (0.33)	1.23 (0.33)	1.24 (0.31)
Triglycerides, mmol/L	1.15 (0.71)	2.04 (1.60)	2.07 (1.73)	1.96 (1.05)
Insulin, mU/L	8.11 (9.81)	17.70 (20.52)	17.81 (16.78)	14.69 (10.47)
ALT, U/L	14.40 (7.89)	26.91 (13.60)	26.57 (13.79)	30.16 (13.94)
AST, U/L	20.57 (5.43)	25.86 (6.99)	25.31 (6.99)	29.35 (7.03)
GT, U/L	24.90 (15.88)	46.66 (23.90)	45.29 (22.95)	58.03 (24.77)
CRP, mg/L	1.39 (2.14)	2.53 (3.24)	2.45 (3.09)	2.81 (3.78)
Alcohol consumption, drinks/day	0.71 (1.01)	1.25 (1.59)	0.65 (0.63)	3.70 (1.95)
Alcohol consumption, g/day	8.50 (12.16)	15.02 (19.13)	7.78 (7.53)	44.38 (23.42)
Physical activity index	9.20 (1.83)	8.40 (1.87)	8.50 (1.84)	8.06 (1.99)

Values are mean (SD).

Note: Alcohol consumption was available from 1,536 subjects. Statistics: Mann-Whitney test was used for continuous parameters and χ2 test for classified parameters when each fatty liver group was compared to controls (NL group). P < 0.001 for all other comparisons, except smoking (FL p = 0.209, NAFL p = 0.548 and AFL p = 0.022) and alcohol consumption (NAFL p = 0.192).

Abbreviations: NL, normal liver; FL, Fatty liver; NAFL, non-alcoholic fatty liver; AFL, alcoholic fatty liver; ALT, alanine aminotransferase; AST, aspartate aminotransferase; GT, glutamyltransferase; CRP, C-reactive protein.

### Gene set enrichment analysis for FL

Fourteen gene sets were upregulated (FDR < 0.05) in subjects with FL (Table 2**)** in Model 1 (adjusting with sex, age, 20 principal components). These gene sets can be grouped into four groups describing the origin of the pathways: (1) pathways related to the extracellular matrix (ECM) turnover, (2) pathways regulating immuno-inflammatory response, (3) pathways contributing to thrombosis and (4) pathways related to neural tissues.

**Table 2:** Gene sets upregulated in subjects with FL compared to NL using GSEA and adjusting for sex, age and 20 principal components (Model 1).

Gene set name	SIZE	ES	NES	Nominal P value	FDR Q value
*ECM turnover*					
INTEGRIN_A4B1_PATHWAY	25	0.66	2.22	<0.001	0.003
INTEGRIN_CELL_SURFACE_INTERACTIONS	47	0.61	2.22	<0.001	0.002
ECM_RECEPTOR_INTERACTION	36	0.59	1.98	<0.001	0.031
MCALPAIN_PATHWAY	17	0.69	2.02	<0.001	0.029
*Immune response regulation*					
CD40_PATHWAY	25	0.65	2.10	<0.001	0.010
LEUKOCYTE_TRANSENDOTHELIAL_MIGRATION	75	0.51	2.04	<0.001	0.025
SIGNALLING_TO_ERKS	28	0.57	1.93	<0.001	0.049
HIVNEF_PATHWAY	54	0.49	1.93	0.002	0.046
*Thrombosis*					
INTEGRIN_ALPHAIIB_BETA3_SIGNALING	20	0.72	2.16	<0.001	0.004
PLATELET_AGGREGATION_PLUG_FORMATION	25	0.75	2.26	<0.001	0.003
UPA_UPAR_PATHWAY	23	0.65	2.00	0.004	0.035
*Neural tissue*					
NETRIN1_SIGNALING	20	0.64	2.02	<0.001	0.026
AGR_PATHWAY	20	0.66	1.98	<0.001	0.034
ALPHA_SYNUCLEIN_PATHWAY	26	0.57	1.89	0.004	0.049

Gene sets with FDR < 0.05, no gene sets were enriched (FDR < 0.05) for downregulation in subjects with FL. Abbreviations: NL, normal liver; FL, fatty liver; GSEA, gene set enrichment analysis; ES, enrichment score; NES, normalized enrichment score; FDR, false discovery rate.

Pathways related to the extracellular matrix (ECM) turnover include two different integrin pathways (integrin A4B1 and integrin cell surface interactions), ECM receptor interaction and mCalpain pathway. All these gene sets share several integrin genes that mediate cell adhesion to ECM.

CD40/CD40L signaling pathway related to immune response was strongly enriched. Also, leukocyte transendothelial migration gene set associated to increased inflammation procedures in individuals with FL. Releated to these inflammation mechanisms, a pathway containing genes involved in signaling to extracellular signal-regulated kinases (ERKs) was also upregulated in FL group. Another upregulated pathway was HIVNEF, which promotes inflammation by helping CD4 helper T cells to prevent apoptosis.

Three upregulated pathways increase the probability for thrombosis in FL. They include integrin alphaIIB beta3 signaling, platelet aggregation plug formation and urokinase plasminogen activator (UPA) – UPA receptor (UPAR) pathway.

Interestingly, some of the upregulated pathways we identified in FL individuals have originally been identified from neural tissues. Netrin-1 signaling pathway, was initially linked to axon development and was recently also associated with atherosclerosis, controlling the trafficking of monocytes between blood and plaque^26^. Another upregulated gene set was Agrin in postsynaptic differentiation (AGR) pathway, which is related to neuromuscular junctions and organization of cytoskeleton in skeletal muscle. Alpha-synuclein signaling pathway, is previously identified from presynaptic neurons in human brain.

With fully adjusted Model 2, four gene sets remained significant (nominal p < 0.05) in FL group **(**Table 3**)**. One of them was related with ECM turnover (integrin A4B1 signaling), two gene sets were related with immune response regulation (CD40/CD40L signaling pathway, leukocyte transendothelial migration) and one gene set associated with neuroimmune guidance cue 1 (netrin-1) signaling.

**Table 3:** Upregulated gene sets with different adjustment models for covariates.

Gene set name	FL (n = 316)		NAFL (n = 223)		AFL (n = 55)	
	M1	M2	M1	M2	M1	M2
*ECM turnover*						
INTEGRIN_A4B1_PATHWAY	<0.001	**0.013**	<0.001	**0.012**	0.820	*NA*
INTEGRIN_CELL_SURFACE_INTERACTIONS	<0.001	0.289	<0.001	0.768	0.025	0.102
ECM_RECEPTOR_INTERACTION	<0.001	0.370	0.002	*NA*	0.132	0.674
MCALPAIN_PATHWAY	<0.001	0.076	0.004	0.089	0.168	0.542
*Immune response regulation*						
CD40_PATHWAY	<0.001	**<0.001**	<0.001	**<0.001**	0.075	0.376
LEUKOCYTE_TRANSENDOTHELIAL_MIGRATION	<0.001	**0.023**	0.016	0.092	0.203	0.369
SIGNALLING_TO_ERKS	<0.001	0.054	0.002	0.082	0.088	0.271
HIVNEF_PATHWAY	0.002	0.080	0.004	0.215	0.042	0.191
*Thrombosis*						
INTEGRIN_ALPHAIIB_BETA3_SIGNALING	<0.001	0.227	0.006	0.926	<0.001	0.119
PLATELET_AGGREGATION_PLUG_FORMATION	<0.001	0.185	0.004	0.898	<0.001	0.139
UPA_UPAR_PATHWAY	0.004	0.107	0.006	0.098	0.017	0.307
*Neural tissue*						
NETRIN1_SIGNALING	<0.001	**0.018**	<0.001	**0.012**	0.202	*NA*
AGR_PATHWAY	<0.001	0.110	<0.001	0.133	0.336	*NA*
ALPHA_SYNUCLEIN_PATHWAY	0.004	0.479	<0.001	0.393	0.242	0.959

Values are shown as nominal p values for each gene set in the specified groups compared to normal liver group (n = 1334).

Statistics: Model 1 (M1) is adjusted for age, sex and 20 principal components; Model 2 (M2) is adjusted for age, sex, 20 principal components, body mass index, apolipoprotein B, triglycerides, insulin, systolic blood pressure, ALT, alcohol consumption and physical activity index. FL, Fatty liver; NAFL, non-alcoholic fatty liver; AFL, alcoholic fatty liver. *NA* means that the pathway was not upregulated in the specific group.

We also present the results of FL vs. NL comparisons for Model 1 in Supplementary Table 1 and for Model 2 in Supplementary Table 2 with cutoff value FDR < 0.25.

### Gene set enrichment analysis with fully adjusted model for NAFL and AFL

All the 14 gene sets upregulated in FL group with Model 1 were also significantly upregulated in NAFL group in comparison to controls (nominal p < 0.05), using Model 1 adjustments. With Model 2 adjusting, we identified three upregulated gene sets: integrin A4B1 signaling, CD40/CD40L signaling pathway and netrin-1 signaling (Table 3**)**.

However, the corresponding results for AFL in Model 1 analysis indicated five of the defined gene sets were significantly enriched for upregulation. They consist of integrin cell surface interactions, HIVNEF pathway and all three thrombosis related pathways (integrin alphaIIB beta3 signaling, platelet aggregation plug formation and UPA–UPAR pathway). For Model 2 analysis with AFL, none of the defined 14 gene sets was significantly upregulated.

When we compared the results of fully adjusted Model 2 with the Model 1, only a few gene sets remained statistically significantly upregulated for FL or NAFL. For example, all pathways related to thrombosis could be explained by the confounding factors. Three pathways were upregulated also in Model 2 analysis of FL and NAFL: integrin A4B1 signaling, CD40/CD40L signaling pathway and netrin-1 signaling pathway.

## Discussion

This large study with 1,650 participants is to our knowledge the first wide-scale peripheral blood based GSEA analysis of subjects with FL. We hypothesized that hepatocellular accumulation of fatty acids and triglycerides trigger an inflammatory response in liver, which is visible via differential immuno-inflammatory gene pathway expression in blood. Furthermore, we investigated the effect of alcohol consumption history and fully adjusted model with previously known independent confounding factors to the observed gene pathways. After adjusting for the known confounding factors, we identified four activated pathways associated with FL, including integrin A4B1 signaling, leukocyte transendothelial migration (TEM), CD40/CD40L and netrin-1 signaling pathways.

These pathways are related to the following biological leukocyte functions: (a) their adherence to vessel wall, (b) transmigration, and/or (c) retaining in tissues. CD40/CD40L signaling pathway is suggested to increase adherence of CD40 positive cells, such as platelets and monocytes, to the vessel wall^27^. Integrin A4B1 (also known as very late antigen-4, VLA-4) signaling pathway is thought to be activated by chemotactic agents from injured cells^28^. Together integrin A4B1 (VLA-4) and leukocyte TEM pathways regulate the leukocyte migration from blood to organs through endothelium^29,30^. Netrin-1 signaling pathway increase leukocyte sensitivity to anti-inflammatory netrin-1, which is shown to decrease the accumulation of neutrophils but is downregulated in injured hepatic tissue^31^. As a consequence of these mechanisms, leukocytes increasingly transmigrate from blood and retain in injured liver tissue. Accumulated lymphocytes are known to induce fatty liver in different ways, such as by activating pro-inflammatory cytokines^8^. Furthermore, activated inflammatory mechanisms increase accumulation of lipids into liver, which then induce or exacerbate the progression of fatty liver. In mouse model of NASH, inflammatory cells also regulate hepatic lipase and lipoprotein lipase: CD8^+^ T cells, NKT cells and cytokines secreted by them induce liver damage, ultimately advancing towards hepatocellular carcinoma^32^. Our present findings would propose background mechanisms behind these previously known pathological changes in FL.

In the present study, upregulated CD40/CD40L signaling pathway was associated with FL, especially NAFL. Sookoian *et al*. found that circulating levels of soluble CD40 ligand (sCD40L) was significantly higher in 113 NAFLD patients when compared to 102 control subjects^33^. This result is in line with our present findings. Previous results by Ercin *et al*. controversially indicated that sCD40L levels did not differ between 50 NAFLD subjects and 30 healthy controls^34^. However, that evaluation was performed with smaller number of subjects than the previous and the present study. CD40L deficiency has been investigated in rodent models as well. Interestingly, Poggi *et al*. found that both the genetic CD40L deficiency and neutralizing anti-CD40L antibody therapy attenuated the development of FL and other metabolic disorders in mice^35^. Based on these previous studies and the present results we would suggest that C40/CD40L signaling pathway plays an important role in metabolic disorders such as FL. More specifically, CD40/CD40L signaling is suggested to increase adherence of CD40 positive cells, such as platelets and monocytes, to the vessel wall modulating atherothrombosis^27^.

Integrin A4B1 signaling pathway was significantly enriched in FL and NAFL groups. As previously indicated, integrin A4B1 (VLA-4) is normally expressed on leukocyte plasma membranes. Activation of this antigen requires chemotactic agents from the site of injury i.e., endothelium or other cells. Endothelium expresses vascular cell adhesion molecule-1 (VCAM-1) which binds to the VLA-4 and enables the transmission of leukocytes from blood to organs^28^. A4B1 has also been shown to bind osteopontin, while facilitating hepatic neutrophil infiltration and liver injury in the rat alcoholic steatohepatitis model^36^. Another significantly enriched pathway after adjusting for confounding factors was leukocyte transendothelial migration (TEM). That pathway is close to the VLA-4 pathway since they both regulate the migration of leukocytes from blood to tissue through endothelium^29,30^. These gene sets correspond to the similar biological functionality but the TEM pathway reflects more biological functions by 75 genes (leukocyte – endothelium cell interactions) while integrin A4B1 signaling targets only 25 genes (leukocyte internal signaling). While Banerjee *et al*. associated leukocyte TEM functions with rat alcoholic steatohepatitis model^36^, we observed that the TEM pathways were activated in NAFL group. However, these pathways may reflect common pathogenic inflammatory mechanisms behind both AFLD and NAFLD^8,9^, and it is possible that the number of subjects with AFL (n = 55) is insufficient to reveal this change in the present study. To determine if there exists difference in TEM pathways between NAFL and AFL in humans, further research is recommended with more subjects in the AFL group. To conclude, in the present study fatty liver is reflected to blood gene pathways in such way where specific factors (adherence and transmigration) driving leukocytes to tissues are activated.

Neuroimmune guidance cue netrin-1 signaling pathway was upregulated in FL. Initially, netrins were considered as cell and axon guidance cue proteins in embryogenesis, and later identified outside the nervous system influencing tissue morphogenesis^37^. Interestingly, in the present study netrin-1 gene was not differentially expressed in our groups. Corresponding results have earlier been observed in another study, where expression of netrin-1 associated with atherosclerotic plaques, but did not differ in blood of patients with coronary artery disease compared with healthy controls^26^. Activated netrin-1 signaling pathway could indicate increased sensitivity of leukocytes and macrophages to the inflammatory mediators such as netrin-1. A recent study with mice suggests netrin-1 as an anti-inflammatory factor in liver ischemia/reperfusion injury^31^, whereas our results extend that also into blood of patients with fatty liver. Netrin-1 is suggested to retain macrophages in atherosclerotic plaques^26^, and by the present results, we would suggest the corresponding effect in patients with FL.

To conclude, these pathways strongly suggest activation of immunological and inflammatory processes in blood of patients with fatty liver. A blood cascade including three different steps was identified with association of fatty liver. Firstly, adherence and transmigration of leukocytes from blood to tissues such as liver and vessel wall is activated. Secondly, interaction of monocytes and platelets is promoted which increases prothrombotic state. Thirdly, netrin-1 signaling pathway induces anti-inflammatory mechanisms by retaining leukocytes into tissues. However, in the present study setting we cannot determine the causality, whether the FL participates in production of these inflammatory factors into blood or do the dysregulated gene pathways refer to systemic disturbances from which FL is one result. Therefore, we are unable to evaluate whether the activation of inflammation system in liver or blood is more important for FL. At least, our results may reflect common metabolic and inflammatory disturbances behind atherosclerosis and FL. Corresponding metabolic results have been identified in a study by Wree *et al*., where increased adipocyte size associated with elevated liver enzymes^38^. In addition, Kälsch *et al*. observed that in population based Heinz Nixdorf RECALL Study increasing liver enzymes (transaminases) even within normal ranges were significantly associated with metabolic changes, i.e., HbA1c, BMI and Type 2 diabetes^39^. These observations support the present results of metabolic disturbances associated with FL. Interestingly, fatty liver status shared several pathways also associated with atherosclerosis or atherothrombosis (CD40/CD40L signaling pathway, Netrin-1 signaling and pathways contributing to thrombosis) i.e., another lipid related condition where lipids are accumulated to artery wall. Furthermore, recent research results would also suggest to link integrin A4B1 to atherosclerosis, where macrophages activate integrin A4B1 by expression of kindlin-3^40^ in atherosclerotic plaques^41^. These shared pathways may form a common link between these two disease states where fatty liver is shown to increase the risk of cardiovascular diseases^5^. This suggestion is also supported by Baars *et al*., who found that elevated liver parameters are associated with stenosis diameter in acute myocardial infarction^42^.

### Limitations and strengths of the study

A strength of the present study is that the subjects are based on the well-powered prospective randomly selected population based cohort, followed up for over 30 years with long well-known history of alcohol use and disease history. This study had large cohort (n = 1,650) and this adds the confidence to the present results. In addition, we used strict FDR limit in GSEA results and heavily adjusted our analysis with known fatty liver risk factors. The original suggestion was FDR < 0.25 but we used FDR < 0.05 as a limit. The strict FDR limit combined to the large number of subjects and adjustment with known risk factors makes the results of this study reliable.

Limitations of the study include population homogeneity: all subjects are Finnish, ethnically homogenous Caucasian people. Because of that these results can be only generalizable in Caucasians. We unfortunately had no possibility for validation of the results in other comparable cohort. Therefore, we recommend the replication of our results and corresponding analysis for further research. In addition, majority of individuals with FL in our population are men. Even though sex has been included in statistical analyses as covariate and sex stratified analyses were performed, possible features specific for FL in women could have been missed due to low number of women with FL. The results cannot therefore be directly generalized to older populations with equal amounts of women and men with FL. The number of subjects in AFL group limited (n = 55), which is probably insufficient to reveal the alterations in the expression of gene pathways in this subgroup. Subjects with FL had more metabolic disorders, which may influence to the present results.

Furthermore, there is a risk of complications when the liver biopsy is taken and it is therefore unacceptable (unethical) to take liver biopsies from healthy asymptomatic individuals on population based studies. Analyzing blood samples is much safer and thus makes it possible to have much larger cohorts. Thus, we decided to analyze gene expression from blood tissue, which restricts the present observations to extrahepatic changes.

The key concept of analyzing gene sets instead of separate genes is considered as a strength of this study. The blood gene pathways reflect real metabolic and systemic functions. We were able to reveal much broader biological mechanisms related to the FL using metabolic pathways instead of single genes.

In conclusion, FL was associated with blood gene sets of inflammatory response, immune system activation and prothrombotic state. These activated blood pathways may serve as a systemic link between FL and the development of cardiovascular diseases.

## Electronic supplementary material

Supplementary information
